# Demoralization in breast cancer survivors

**DOI:** 10.3389/fpsyg.2025.1523164

**Published:** 2025-07-11

**Authors:** Annekathrin Sender, Andreas Hinz, Laura Broemer, Anja Mehnert-Theuerkauf, Bernhard Strauß, Susanne Briest, Jenny Rosendahl

**Affiliations:** ^1^Department of Medical Psychology and Medical Sociology, University Medical Center Leipzig, Comprehensive Cancer Center Central Germany (CCCG), Leipzig, Germany; ^2^Institute for Psychosocial Medicine, Psychotherapy and Psychooncology, University Hospital Jena, Jena, Germany; ^3^Department of Gynecology, University Medical Center Leipzig, Leipzig, Germany

**Keywords:** demoralization, breast cancer, demoralization scale, religiousness, spirituality, meaning, mental health, cancer survivors

## Abstract

**Objective:**

Demoralization is a state of existential distress and loss of meaning in life and often associated with poor quality of life in cancer survivors. This study aimed to test psychometric properties of the Demoralization Scale-II (DS-II), to compare demoralization in breast cancer survivors with scores obtained in the general population in Germany, and to identify prognostic factors for demoralization and associations with spiritual well-being and other related variables.

**Methods:**

We analyzed a sample of 162 female breast cancer survivors (mean time since diagnosis 2.5 years) using the DS-II and a set of validated questionnaires measuring distress (DT), anxiety (GAD-7), depression (PHQ-9), fatigue (MFI-10), spiritual well-being (FACIT-Sp) posttraumatic growth (PTGI) as well as meaning and purpose (LAP-R). The DS-II mean scores of the survivor’s sample were compared with those of the general population. We performed t-tests, effect sizes, Cronbach’s alpha, correlations and a confirmatory factor analysis to achieve the study objectives.

**Results:**

The DS-II showed excellent psychometric properties, with an internal consistency of *α* = 0.92 for the total scale and α = 0.88 for the meaning and purpose subscale as well as α = 0.85 for the distress and coping subscale. Demoralization was highly correlated with the experience of meaning and peace (*r* = –0.79), sense of existential vacuum (*r* = −0.78), anxiety (*r* = 0.72), and depression (*r* = 0.70), while the correlations were lower for distress (*r* = 0.37) and post-traumatic growth (*r* = −0.19). The mean scores of the breast cancer survivors were markedly higher than those of the general population on both subscales distress and coping (*d* = 0.71) and meaning and purpose (*d* = 0.34).

**Discussion:**

The results show that demoralization is prevalent in cancer survivors and should be understood as a proper construct that has multiple relationships with other variables of mental health. This underlines the need to establish evidence-based support programs that focus on demoralization and address these links.

## Introduction

Demoralization is a state of existential distress that is characterized by feelings of hopelessness, helplessness, subjective incompetence and loss of meaning and purpose (Clarke and Kissane, 2002; Robinson et al., 2015; Mesquita Garcia et al., 2023; Frank, 2010; de Figueiredo and Frank, 1982), recently often described as “Demoralization Syndrome.” A recent systematic review found prevalence rates across diverse clinical settings of up to 36% (Gan et al., 2022). In oncology and palliative care in particular, patients are confronted with a life-threatening diagnosis, which can lead to an increased level of demoralization (Fava et al., 2023; Oberth et al., 2024). The prevalence of demoralization in cancer patients ranges from 13.50 to 49%, with a mean prevalence of 36% (Wang et al., 2023). In samples of mixed cancer patients with advanced-stages, demoralization has been found to range up to 52% (Robinson et al., 2015; Bovero et al., 2023; Ignatius and De La Garza, 2019). Demoralization is strongly associated with depression (Lin et al., 2022; Bobevski et al., 2022a), anxiety and death anxiety (An et al., 2018; Robinson et al., 2015), existential distress (Bovero et al., 2018), low levels of quality of life (Chang et al., 2022; Bovero et al., 2023), unfavorable relationships with health care providers (Quintero Garzón et al., 2018) and family caregivers (Bovero et al., 2022; Bovero et al., 2023), wish to die (Belar et al., 2021), and suicidal ideation (Vehling et al., 2017).

From a conceptual point of view, demoralization can be understood as the opposite of spiritual well-being (Mesquita Garcia et al., 2023; Almeida et al., 2022), a notion that is also reflected in related research. Several studies found negative associations between demoralization and spirituality (Ghiggia et al., 2021), hope (Airoldi et al., 2018), and posttraumatic growth (Li et al., 2015). However, the results were inconclusive regarding the relationship between religion and demoralization. A recent systematic review found inverse relationships between demoralization and spirituality. One Chinese study, though, found higher levels of demoralization in religious patients (Shao et al., 2024). Another Chinese study failed to detect associations between demoralization and religion (Ko et al., 2018). In contrast, a study with Spanish-speaking patients reported lower levels of demoralization in patients performing religious practices.

Many of the studies on demoralization have been performed with advanced cancer patients and other terminally ill patients (Vehling et al., 2015; Bovero et al., 2018; Bovero et al., 2023; Julião et al., 2016). However, studies focusing on cancer survivors, particularly breast cancer survivors, are rare and there is therefore a need for further studies examining demoralization and its associations with other variables in this patient group. Moreover, there is a lack of studies that compare the demoralization scores of cancer survivors with scores obtained in the general population.

To assess demoralization, several instruments have been applied, such as the Demoralization Scale (DS, Kissane et al., 2004), the Demoralization Interview (DI, Bobevski et al., 2022b) the Diagnostic Criteria for Psychosomatic Research Semi-Structured Interview (DCPR-SSI, Guidi and Fava, 2022), and the Subjective Incompetence Scale (SIS, Cockram et al., 2009). While most instruments were originally developed for and validated in medical populations—especially cancer and other severe illnesses— recent research has extended their use to broader populations (Tecuta et al., 2015; Fava and Guidi, 2023). A frequently used instrument within these for assessing demoralization is the Demoralization Scale. In its original form (DS-I), this instrument comprised 24 items that were assigned to five dimensions. A revised and shortened form of this questionnaire is the 16-item Demoralization Scale DS-II with 16 items and two subscales: (1) Meaning and Purpose as well as (2) Distress and Coping (Robinson et al., 2016a; Robinson et al., 2016b). Several studies have already been performed to test psychometric properties of this DS-II scale (Robinson et al., 2016b; Belar et al., 2019; Elmasian et al., 2023; Koranyi et al., 2021). Normative values have been published for the DS-I (Quintero Garzón et al., 2021) and the DS-II as well (Ramm et al., 2023). This enables a comparison of item and subscale mean scores obtained in samples of cancer patients or survivors with those of the general population. Such comparisons have not be published so far, but they have the potential to give a deeper insight in the components of demoralization that determine the difference between cancer survivors and individuals in the general population.

The objectives of this study were (a) to test psychometric properties of the DS-II, applied to breast cancer survivors, (b) to determine the degree of demoralization in breast cancer survivors in comparison with the general population, (c) to test whether sociodemographic factors including religious affiliation determine the degree of demoralization, and (d) to examine the relationship between demoralization and spiritual well-being as well as associations with anxiety, depression, distress, and fatigue.

## Methods

### Sample of breast cancer survivors

The present data were collected as part of a cross-sectional, bi-center study in which female breast cancer patients were surveyed on the topic of sense of meaning, including the assessment of demoralization and other related variables. Adult breast cancer survivors were enrolled from the hospital documentation systems of two University Medical Centers (Leipzig and Jena) in central Germany. Both hospitals serve urban and rural areas, with a large proportion of patients from the surrounding rural region. Individuals were eligible for study participation if they were (a) female; (b) ≥ 18 years old at the time of study inclusion; (c) able to speak und read German and (d) had a confirmed initial diagnosis of stage I to III breast cancer within the previous 5 years with completed primary medical cancer treatment. Those with severe verbal, physical and/or cognitive impairments that interfered with their ability to give informed consent were excluded. We also excluded patients diagnosed as stage IV according to UICC classification because their likelihood of survival is significantly reduced.

Eligible survivors were identified by a trained study research assistant via the clinic patient database in both centers. We successfully recruited from 2020 to 2021 over a 12 months period. We contacted eligible survivors by mail. They received a study information letter, a letter of consent and the paper-pencil based questionnaire to complete at home. The letter included the study team’s contact details, and participants were encouraged to contact the study team in order to clarify any questions relating to the study. The study protocol was approved by the local ethics committee (reference number 2021-2106-Bef). All participants provided written informed consent prior to study participation.

### Measures

#### Sociodemographic and clinical characteristics

Sociodemographic information, i.e., age, education, employment status, sick leave, relationship status, perceived religiousness/spirituality and religious affiliation, was collected via self-report within the questionnaire. Medical information, i.e., diagnosis, date of diagnosis and information on treatment, was extracted from medical charts.

### Questionnaires

#### DS-II

Demoralization was assessed with the Demoralization Scale-II (DS-II) (Robinson et al., 2016a; Robinson et al., 2016b). It is a shortened and modified version of the DS-I (Kissane et al., 2004) and consists of 16 items scored on a 3-point Likert scale ranging from “never” (0) to “often” (2). The instrument contains two 8-item subscales: (1) Meaning and Purpose, and (2) Distress and Coping. Sum score values range from 0 to 32 with higher scores reflecting higher levels of demoralization. The total scores can be assigned to three categories: low (0–3), moderate (4–10), and high (≥ 11) demoralization (Robinson et al., 2016a). Recently, norm values of the DS-II have been published (Ramm et al., 2023).

#### FACIT-Sp

Spiritual well-being was measured with the Functional Assessment of Chronic Illness Therapy - Spirituality Scale (FACIT-Sp) (Peterman et al., 2002). The FACIT-Sp is a 12-item questionnaire scored on a 5-point Likert scale with higher scores representing greater spiritual well-being. The scale consists of two dimensions: Meaning/Peace (8 items) and Faith (4 items) (Damen et al., 2021; Aktürk et al., 2017).

#### PTGI

The Posttraumatic Growth Inventory (PTGI) (Maercker and Langner, 2001) consists of 21 items that are assigned to five dimensions: Relating to others (7 items), New possibilities (5 items), Personal strength (4 items), Spiritual change (2 items), and Appreciation of life (3 items). All items are rated on a 3-point Likert scale. Higher scores indicate higher degrees of posttraumatic growth (Mostarac and Brajković, 2022).

#### LAP-R

The Life Attitude Profile-Revised (LAP-R) (Mehnert and Koch, 2008) is a 25 item instrument for measuring meaning and purpose in life. It comprises six subscales: Purpose (1 item), Coherence (5 items), Choice/responsibleness (5 items), Death acceptance (5 items), Existential vacuum (4 items) and Goal seeking (5 items). All items are scored on a 7-point Likert scale. Higher scores indicate higher degrees of meaning and purpose in life except for the subscales Existential vacuum and Goal seeking (Valdés-Stauber et al., 2021).

#### MFI-10

Fatigue was assessed with the Multidimensional Fatigue Inventory-10 (MFI-10) (Baussard et al., 2018). The questionnaire comprises 10 items with five response options each. The subscales are Physical fatigue (4 items), Emotional fatigue (4 items), and Cognitive fatigue (2 items). Higher scores indicate higher levels of fatigue for each subscale.

#### DT

Distress was recorded with the NCCN Distress Thermometer (DT) (Mehnert et al., 2006; Sun et al., 2022). The DT contains a single-item visual analog scale ranging from 0 to 10 to quantify the psychological symptom burden during the past week. Higher scores indicate higher mental distress. The scale is recommended to be routinely used in clinical cancer care and a cut-off score of 5 is used to identify clinical significant distress (National Comprehensive Cancer Network, 2003).

#### PHQ-9

Depression was assessed with the Patient Health Questionnaire (PHQ-9) (Kroenke et al., 2001). The nine items refer to the frequency of depressive symptoms within the past 2 weeks. All items are scored on a 4-point Likert scale. Higher scores represent higher depressive symptomatology with a score of 5–9 indicating mild depression, 10–14 moderate depression, 15–19 moderately severe depression, and 20–27 severe depression. A score of 10 or higher is commonly used as the threshold for clinically significant depressive symptoms.

#### GAD-7

Anxiety was assessed with the Generalized Anxiety Disorder Screener (GAD-7) (Spitzer et al., 2006; Löwe et al., 2008). The GAD-7 comprises seven items related to the frequency of core symptoms of a generalized anxiety disorder over the previous 2 weeks. As with the PHQ-9, all items are scored on a 4-point Likert scale, and higher scores indicate higher levels of anxiety. A score of 10 or higher is used as the cut-off for clinically significant anxiety symptoms and scores of 5–9 indicate mild anxiety, 10–14 moderate anxiety, and 15–21 severe anxiety.

*Perceived religiousness/spirituality* was assessed with one question that included five response options, ranging from not at all (1) to very much (5). In addition, the women were asked whether they were affiliated with a religious organization (protestant/catholic/other).

### Statistical analysis

IMB SPSS Statistics 29 was used for statistical data analyses. T-tests were conducted to test mean core differences between subgroups of the sample, and Cohen’s *d* was used to express the effect size of the group comparison.

Data from a representative general population sample (Ramm et al., 2023) were used to compare the breast cancer survivor’s data with. In that publication, mean values of the scales of the DS-II are given separately for gender and age groups, but the mean values of the items are only listed for the overall sample (Ramm et al., 2023). In order to enable a fair comparison between cancer survivors and the general population at item level, the following procedure was chosen: At scale level, we compared the values of the group of the female cancer survivors with the subgroup of women aged 50–59 from the representative sample, as this group corresponds best to the patient group. Within the general population sample, women in the age range 50–59 years scored higher (total score: *M* = 4.44) than the general population as a whole (*M* = 3.76), with an effect size of *d* = 0.12. For each item, we performed a linear transformation so that the difference between the total sample of the general population and the group of women aged 50–59 years was considered, applying the effect size of *d* = 0.12 to all items. This procedure slightly reduced the comparison values of the general population for each item in order to enable a fair comparison between the cancer survivors’ sample and the general population at item level as well. The standard deviations were transformed in the same way.

A confirmatory factor analysis (CFA) was conducted to test the one factor model and the two-factor model of the DS-II, applying the coefficients Comparative Fit Index (CFI), Tucker-Lewis-Index (TLI), Root Mean Square Error of Approximation (RMSEA), and Standardized Root Mean Squared Residual (SRMR). Pearson correlations were calculated to describe associations of demoralization with several measures of psychological symptom burden, meaning, and posttraumatic growth.

## Results

### Sample characteristics

Out of 395 eligible cancer survivors, 162 participated in the study (response rate 41%). In the responder analysis, the study participants were compared with the non-participants. There were no significant differences between the two groups in terms of age, diagnosis, time since diagnosis and stage of disease. Sociodemographic and clinical characteristics are presented in Table 1. The mean age was 56.0 years (SD = 11.1), and the mean time since initial breast cancer diagnosis was 2.5 years (SD = 1.4 years, range 1.3–17.4 years).

**Table 1:** Sociodemographic and clinical characteristics.

Sociodemographic and clinical variables	Total (*n* = 162)	
	*n*	%
Age		
< 60 years	90	55.5
≥ 60 years	72	44.4
Education^(a)^		
< 12 years	81	50.3
≥ 12 years	80	49.7
Employment status^(a)^		
Unemployed	77	48.4
Employed	82	51.6
Sick leave^(a)^		
On sick leave	7	4.4
Not on sick leave	153	95.6
Relationship status^(a)^		
Single	31	19.6
In a relationship	127	80.4
Time since diagnosis^(a)^		
≤ 2 years	62	38.5
> 2 years	99	61.5
Perceived religiousness/spirituality		
Low	119	73.5
Medium to strong	43	26.5
Religious affiliation^(a)^		
None	128	81.0
Protestant/Catholic/Other	30	19.0

^(a)^Missing values not reported.

### Psychometric properties and item analyses of the DS-II

Table 2 presents item and scale characteristics of the DS-II. The item mean scores ranged from 0.17 (I would rather not be alive) to 1.01 (I tend to feel hurt easily). All part-whole-corrected item-test correlations were between 0.47 and 0.79. The item with the highest correlation was item 7 (I feel hopeless). The internal consistency of the total scale was *α* = 0.92, while α values of the two subscales were somewhat lower (0.88 and 0.85, respectively), see Table 2. Both subscales of the DS-II correlated with each other at 0.775.

**Table 2:** Item and scale characteristics (DS-II) of the survivors’ group and comparisons with the general population.

Item		Sub-scale	Survivors					General population		Surv. - Gen. Pop.
			M	SD	*r*_it_ sub-scale 1	*r*_it_ sub-scale 2	*r*_it_ total scale	M	SD	*d* (Surv., Gen. Pop.)
1	There is little value in what I can offer to others.	1	0.57	(0.60)	0.47		0.49	0.58	(0.71)	−0.01
2	My life seems to be pointless.	1	0.30	(0.52)	0.72		0.66	0.20	(0.48)	0.20
3	My role in life has been lost.	1	0.30	(0.57)	0.71		0.70	0.25	(0.54)	0.09
4	I no longer feel emotionally in control.	2	0.52	(0.63)		0.55	0.62	0.21	(0.47)	0.56
5	No one can help me.	1	0.46	(0.64)	0.68		0.68	0.21	(0.49)	0.44
6	I feel that I cannot help myself.	1	0.52	(0.61)	0.71		0.74	0.25	(0.52)	0.48
7	I feel hopeless.	1	0.39	(0.59)	0.79		0.79	0.20	(0.48)	0.35
8	I feel irritable.	2	0.86	(0.67)		0.55	0.52	0.47	(0.63)	0.60
9	I do not cope well with life.	2	0.39	(0.57)		0.64	0.75	0.22	(0.52)	0.31
10	I have a lot of regret about my life.	2	0.69	(0.64)		0.55	0.53	0.38	(0.63)	0.49
11	I tend to feel hurt easily.	2	1.01	(0.70)		0.61	0.57	0.38	(0.60)	0.97
12	I feel distressed about what is happening to me.	2	0.75	(0.65)		0.49	0.49	0.31	(0.58)	0.72
13	I am not a worthwhile person.	1	0.24	(0.51)	0.62		0.62	0.18	(0.48)	0.13
14	I would rather not be alive.	1	0.17	(0.41)	0.57		0.51	0.08	(0.34)	0.23
15	I feel quite isolated or alone.	2	0.38	(0.59)		0.62	0.66	0.27	(0.57)	0.19
16	I feel trapped by what is happening to me.	2	0.44	(0.65)		0.65	0.68	0.25	(0.55)	0.32
	Subscale 1		2.95	(3.33)	α = 0.88			1.87	(3.10)	0.34
	Subscale 2		5.03	(3.56)		α = 0.85		2.57	(3.35)	0.71
	Total scale		7.98	(6.50)			α = 0.92	4.44	(6.25)	0.56

r_it_: part-whole-corrected item-test correlations; α: Cronbach’s alpha.

The CFA results for the one-factor model were as follows: CFI: 0.891, TLI: 0.875, RMSEA: 0.085, SRMR: 0.062. The corresponding coefficients for the two-factor model were: CFI: 0.917, TLI: 0.903, RMSEA: 0.075, SRMR: 0.058.

The comparison between the mean scores of the study sample and the general population [females, age range 50–59 years according to the normative values (Ramm et al., 2023)] is presented in the right side of Table 2. For all items except one, the survivors’ demoralization mean scores were higher than those of the general population. The item with the highest effect size (*d* = 0.97) was item 11 (I tend to feel hurt easily).

Regarding the two subscales, the effect size of the difference between survivors and the general population was higher in subscale 2 (Distress and coping, *d* = 0.71) than in subscale 1 (Meaning and purpose, *d* = 0.34), and the effect size of the total scale was between these two values (*d* = 0.56). According to the categorization given by the authors of the DS-II, the frequencies of the three categories in the survivors’ group were 31.1% (low), 38.8 (moderate), and 30.0 (high demoralization).

### Associations between sociodemographic factors and demoralization

The impact of sociodemographic factors on demoralization is illustrated in Table 3. The impact of age, education, and relationship status was negligible. Survivors with higher levels of perceived religiousness/spirituality showed significantly higher levels of demoralization than survivors with lower levels (*d* = 0.50 for the total scale). On the level of formal belongingness to a religious institution, this effect was markedly lower (*d* = 0.15) and not statistically significant.

**Table 3:** Means and mean differences of demoralization across demographic groups.

Demographic groups	Meaning and purpose				Distress and coping ability				Total scale			
	*M*	SD	*d*	*p*	M	SD	*d*	*p*	M	SD	*d*	*p*
Age												
< 60 years	2.93	(3.29)	0.01	ns	5.15	(3.73)	−0.08	ns	8.08	(6.58)	−0.04	ns
≥ 60 years	2.98	(3.40)			4.87	(3.34)			7.85	(6.44)		
Education												
< 12 years	3.00	(3.10)	−0.05	ns	4.97	(3.50)	0.02	ns	7.98	(6.12)	−0.01	ns
≥ 12 years	2.85	(3.55)			5.05	(3.64)			7.90	(6.89)		
Employment status												
Unemployed	3.28	(3.53)	−0.20	ns	5.40	(3.69)	−0.21	ns	8.69	(6.93)	−0.21	ns
Employed	2.63	(3.12)			4.67	(3.40)			7.30	(6.03)		
Relationship status												
Single	2.91	(3.14)	0.00	ns	4.63	(3.25)	0.14	ns	7.52	(6.01)	0.08	ns
In a relationship	2.90	(3.38)			5.13	(3.69)			8.04	(6.69)		
Time since diagnosis												
≤ 2 years	3.17	(3.63)	−0.11	ns	5.06	(3.83)	−0.01	ns	8.23	(7.22)	−0.06	ns
> 2 years	2.81	(3.13)			5.01	(3.39)			7.83	(6.03)		
Perceived religiousness/spirituality												
Low	2.49	(3.03)	0.50	**	4.59	(3.29)	0.45	**	7.08	(5.94)	0.50	**
Medium to strong	4.19	(3.79)			6.23	(4.00)			10.43	(7.36)		
Religious affiliation												
None	2.86	(3.29)	0.15	ns	4.85	(3.35)	0.20	ns	7.71	(6.29)	0.19	ns
Protestant/catholic/other	3.38	(3.58)			5.60	(4.06)			8.99	(7.15)		

**p < 0.01, ns: not significant; M: mean; SD: standard deviation; d: effect size.

### Associations between demoralization and other variables

Table 4 presents the associations between the scales of the DS-II and several other scales. Regarding the DS-II total scale, the strongest correlations were found for the FACIT-Sp scale Meaning/peace (*r* = −0.79) and the LAP-R scale Existential vacuum (*r* = −0.78), while the association with the scale Faith of the FACIT-Sp was relatively weak (*r* = −0.17). The total PTGI score was negatively correlated with the DS-II total scale (*r* = −0.19). Distress, as measured with the DT, showed moderate positive correlations with the DS-II scales (DS-II total scale *r* = 0.37), and the total MFI scale for Fatigue moderately to strongly correlations were found with the DS-II scales (DS-II total scale *r* = 0.56). Both depression (PHQ-9) and anxiety (GAD-7) showed strong correlations with all DS-II scales with PHQ-9 correlating with the DS-II total scale at *r* = 0.70 and GAD-7 at *r* = 0.72.

**Table 4:** Correlations between demoralization and other scales.

Scales	DS-meaning and purpose		DS-distress and coping ability		DS-total scale	
FACIT-Sp						
Meaning/peace	−0.77	***	−0.72	***	−0.79	***
Faith	−0.18	*	−0.15	ns	−0.17	*
Total FACIT-Sp	−0.66	***	−0.60	***	−0.67	***
PTGI						
Relating to others	−0.20	*	−0.06	ns	−0.13	ns
New possibilities	−0.21	**	−0.09	ns	−0.16	*
Personal strength	−0.31	***	−0.30	***	−0.32	***
Spiritual change	0.06	ns	0.08	ns	0.07	ns
Appreciation of life	−0.17	*	−0.04	ns	−0.11	ns
Total PTGI	−0.24	**	−0.12	ns	−0.19	*
LAP-R						
Coherence	−0.49	***	−0.28	***	−0.41	***
Existential vacuum	−0.73	***	−0.74	***	−0.78	***
Choice/Responsibleness	−0.44	***	−0.39	***	−0.44	***
Death acceptance	−0.33	***	−0.30	***	−0.33	***
Goal seeking	−0.09	ns	−0.02	ns	−0.06	ns
MFI-10						
Physical fatigue	0.35	***	0.51	***	0.46	***
Emotional fatigue	0.48	***	0.56	***	0.55	***
Cognitive fatigue	0.42	***	0.55	***	0.52	***
Total MFI	0.46	***	0.59	***	0.56	***
DT (distress)	0.33	***	0.37	***	0.37	***
PHQ-9 (depression)	0.61	***	0.71	***	0.70	***
GAD-7 (anxiety)	0.60	***	0.75	***	0.72	***

*p < 0.05; **p < 0.01; ***p < 0.001; ns: not significant.

## Discussion

This study aimed to evaluate the psychometric properties of the DS-II questionnaire, applied to breast cancer survivors, and to analyze their experience of demoralization.

The DS-II demonstrated high levels of internal consistency and reliability, which supports its suitability for use with breast cancer survivors. The internal consistency coefficients *α* of both subscales. Were even somewhat higher than those reported from other studies (Robinson et al., 2016b; Belar et al., 2019; Wu et al., 2021; Palacios Espinosa et al., 2020). This may be because these studies mainly involved patients with advanced cancer who might be restricted in the consistency of their responses to the items of the questionnaire.

All aspects assessed by the DS-II were found to contribute meaningfully to the overall measure of demoralization, with particularly strong associations observed for feelings of hopelessness and difficulties in coping. This highlights that hopelessness is a key component of demoralization in breast cancer survivors. Therefore, in order to impede demoralization, clinicians need to convey hope to their patients: hope for successful therapy and ensuring they feel supported by competent, sensitive caregivers may help counteract demoralization. Additionally, the findings emphasize the importance of patient’s self-efficacy in relation to demoralization. Those who feel unable to help themselves or struggle to cope with daily life appear especially vulnerable. Therefore, interventions that strengthen patients’ self-efficacy and competence to impact the course of the illness and the treatment in a positive way, could improve clinical outcomes and also prevent demoralization to a certain degree. A patient who feels competent may be less likely to feel helpless. Thus, supporting patients in recognizing the importance of health-promoting behaviors and adherence to treatment, could be a valuable strategy in comprehensive survivorship care.

A strong and significant association was observed between the two subscales of the DS-II, suggesting that the different aspects of demoralization are closely related in this population. The results of the CFA showed that neither the one-dimensional model nor the two-dimensional model reached the recommended thresholds for good fit indices, though the coefficients of the two-factor model were somewhat better than those of the one-factor model. These findings are consistent with previous research involving German (Koranyi et al., 2021) and Greek (Elmasian et al., 2023) cancer patient samples, in which similar issues with model fit were reported. Based on the unsatisfactory CFA results, the authors of the Greek study created a new scale structure that achieved more favorable fit values for their data (Elmasian et al., 2023). Although it would have been possible to develop a new structure tailored to our sample, we opted not to take this approach. We believe that maintaining a consistent scale structure across studies is essential for comparability and for building a robust body of evidence in the field of demoralization research. The high internal consistency of the total scale, the strong association between the subscales and the generally higher correlations of the total scale with related constructs all support the continued use of the original DS-II structure.

When comparing breast cancer survivors to the general population, we observed that survivors reported substantially higher levels of demoralization across both dimensions measured by the DS-II. These results replicate those of Koranyi et al. (2021) and Elmasian et al. (2023). This difference was particularly evident in aspects relating to emotional distress and coping difficulties, indicating that these issues are relevant for survivors. In contrast, more spiritual elements related to meaning, purpose and self-esteem showed smaller differences between the two groups. In line with previous research, this pattern suggests that demoralization in the context of breast cancer survivorship is characterized by a heightened vulnerability to distress, and feelings of emotional overwhelm (Wu et al., 2021), as well as a perceived inability to cope with the challenges of the illness and its consequences (Belar et al., 2019). A diminished sense of personal value or loss of life roles, however, appears to play a less central role in survivors’ experience of demoralization (Mesquita Garcia et al., 2023). Supporting survivors in managing emotional distress and enhancing their coping resources may be particularly effective in addressing the most common aspects of demoralization they experience.

Considering the impact of social and demographic factors, age was not significantly associated with demoralization. This is in line with another study that also did not detect age differences (Katz et al., 2001), while there are studies with positive (Vehling et al., 2011) and also negative (Mehnert et al., 2011; Vehling et al., 2013) associations. This means that age does not have a consistent impact on demoralization, and the health status is a more important factor than the age itself. Education was also uncorrelated with demoralization. This finding is consistent with previous studies (Katz et al., 2001; Lee et al., 2012), though other studies reported a positive association between demoralization and education (Koranyi et al., 2021) or a negative association (Ko et al., 2018).

Although unemployed survivors showed higher levels of demoralization than employed ones, this difference was not statistically significant. It is interesting to note that living in a relationship does not seem to have an effect on demoralization in our study. Having a partner and a social network has often been described as protective factors that prevent mental health problems (Okati-Aliabad et al., 2022; Grav et al., 2012; Yoo et al., 2017). However, having a partner may also cause survivors to worry about their situation and the future of their partner, which could cancel out the protective effect.

The only statistically significant group difference was related to perceived religiousness/spirituality. In contrast to expectations, survivors with a high perceived degree of religiousness/spirituality showed relatively high levels of demoralization. The affiliation to a religious community, however, had no significant impact on demoralization. This means that it is necessary to distinguish between the formal affiliation with a religious group and the personally perceived spirituality. Secularization has advanced considerably in Eastern Germany, as reflected in the relatively low percentage of women (19%) who belong to a religious community. Those who are affiliated with a church might experience more consolation and confidence, but it is also possible that their claim to a meaningful life is higher, and therefore, the discrepancy between the expected state of their health and healing is perceived as being higher than in those survivors without church affiliation.

As in our study, an investigation with Chinese cancer patients (Shao et al., 2024) found a positive relationship between religiousness and demoralization, whereas a study with cancer patients in Spanish-speaking countries (Belar et al., 2019) observed a negative association between religious practice and demoralization. Therefore, cultural differences in the role of religious and spiritual attitudes in the development of demoralization have to be taken into account, and that it is important to distinguish between religious practice and perceived spirituality. A recent systematic review (Mesquita Garcia et al., 2023) found that unmet spiritual needs were associated with increased demoralization among cancer patients, while spiritual well-being served as a protective factor against demoralization. This highlights the importance of tending to cancer patients’ spiritual needs, for example in the form of pastoral care or meaning-centered therapy (Vibrans et al., 2023).

The correlations between the scales of the DS-II and the other scales of spiritual well-being and mental health are instructive. Demoralization was strongly associated with existential and spiritual well-being, particularly with regard to meaning, peace, and existential concerns. These associations were stronger than those with anxiety and depression. This is consistent with previous research (Palacios Espinosa et al., 2020; Mesquita Garcia et al., 2023) and indicates that demoralization is more than just an intense form of depression. The unique association with loss of meaning suggests that demoralization should be considered a distinct psychological construct. Although anxiety and depression showed similar strengths of association with demoralization, the relationship with distress was comparatively weak. This may partly reflect limitations in the way the construct was measured (single item), which reduces the reliability. More importantly, it likely indicates that distress—in terms of feeling excessive demands, time pressure or workload—is a common experience, even among healthy individuals, and thus less specific to the demoralization syndrome (Robinson et al., 2016b).

Some limitations of the study should be mentioned. The response rate was relatively low, and the sample might be biased toward a higher proportion of women in a relatively good state of health. It was not possible to report the specific reasons for participants dropping out or not responding. Questionnaire invitations were sent by mail and participation was voluntary. Due to data protection regulations, it was not possible to contact non-respondents. Consequently, no information could be collected regarding the reasons for non-participation or dropping out, which may limit insights into potential response biases. The absence of significant associations between certain sociodemographic characteristics and demoralization may be due to the small number of participants in some subgroups, such as those with a religious affiliation. Therefore, non-significant findings should be interpreted with caution, as they do not necessarily indicate the absence of an effect. With regard to the CFA the sample size was relatively small, so that the fit coefficients should also be viewed with reservation.

While the study included multiple mental health questionnaires, clinical data were limited. Thus, the relationships between clinical variables and demoralization could not be addressed here. All correlations with mental health and spirituality questionnaires may be subject to some response bias (Rammstedt et al., 2017), so Pearson correlations may slightly overestimate the true associations. Since only breast cancer survivors were studied, our findings cannot be generalized to other types of cancer. Similarly, the relationships found between religiousness and demoralization can also be attributed to cultural characteristics, and these conditions may vary across different cultures.

In summary, the DS-II demonstrated good internal consistency and acceptable construct validity, confirming its practicable value for evaluating demoralization in breast cancer survivors. Both subscales of the DS-II capture unique aspects of demoralization—such as hopelessness, distress, meaning and coping difficulties—that are not fully addressed by measures of depression or existential distress, as each DS-II subscale addresses different aspects of demoralization. Maintaining the original DS-II structure ensures comparability across studies and supports its continued use in research and clinical practice. Comparison with normative data highlights that hopelessness is a particularly prominent component of demoralization among breast cancer survivors.

In addition to the correlations with other constructs of mental health, the comparison with the normative data from the general population shows a differentiated picture of the components of demoralization, with an emphasis on the issue of hopelessness. Clinically, this underlines the importance of fostering and maintaining hope as a central part of survivorship care. Demoralization occurs at a considerable level among breast cancer survivors in this sample and is associated with multiple mental health variables. It is closely linked to emotional distress and coping abilities, which emphasize the need for targeted interventions and further research to improve psychosocial support for breast cancer survivors. The current review by Dong et al. (2024) shows that demoralization has rarely been a primary outcome in intervention trials. This highlights the need for RCT studies aimed at developing effective treatments and reducing demoralization in cancer survivors.

## Data Availability

The raw data supporting the conclusions of this article will be made available by the authors, without undue reservation.
